# A Case of Orbital Cellulitis After Hyaluronic Acid Filler Injection: A Case Report

**DOI:** 10.1111/jocd.70530

**Published:** 2025-11-07

**Authors:** Firas F. Haddad, Serena Saade, Rami Abadi, Raed Rtail

**Affiliations:** ^1^ Faculty of Medicine American University of Beirut Beirut Lebanon; ^2^ RA Clinics Beirut Lebanon; ^3^ School of Medicine St. George's University Beirut Lebanon

**Keywords:** cosmetic dermatology, dermatology, fillers, infections, orbital cellulitis

To the Editor,

A 38‐year‐old lady presented to our clinic with fever, pain, blurry vision and significant erythema and swelling of the right side of her face 2 weeks after injection into the cheek with an unknown hyaluronic acid filler by a non‐specialist (Figures 1 and 2). Blood workup was conducted and showed significant leukocytosis with a WBC count of 29 320 with a left shift with neutrophilic predominance (89.7%). The CRP was elevated with a value of 104.9. In light of these laboratory values, the working diagnosis was orbital cellulitis, and a CT scan of the orbit (Figure 2) was conducted and showed soft tissue thickening and fat stranding involving the right side of the face including the temporal region and orbit highly suggestive of cellulitis and orbital cellulitis.

**FIGURE 1:**
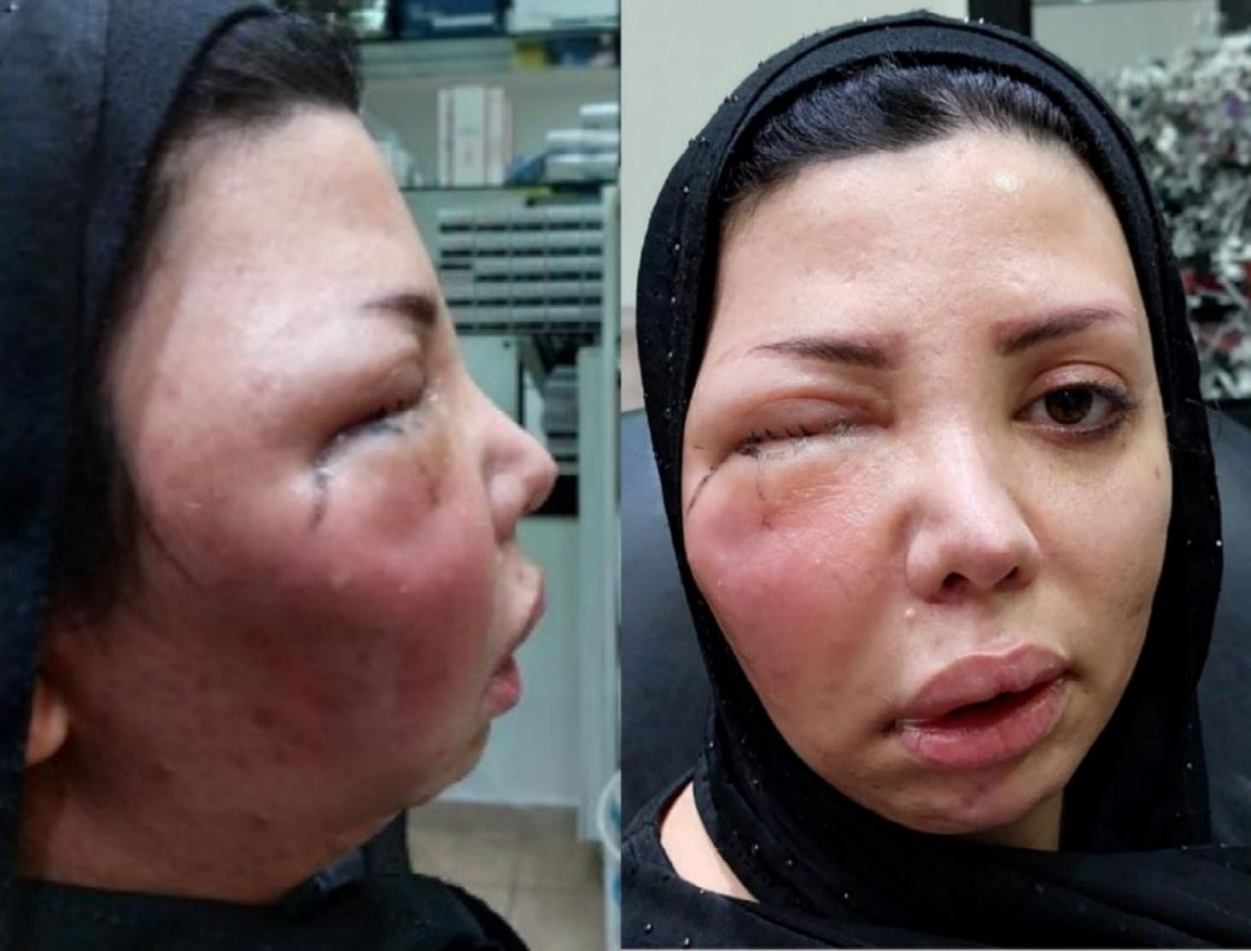
Clinical images showing significant erythema and swelling of the right side of her face 2 weeks after injection with hyaluronic acid filler.

**FIGURE 2:**
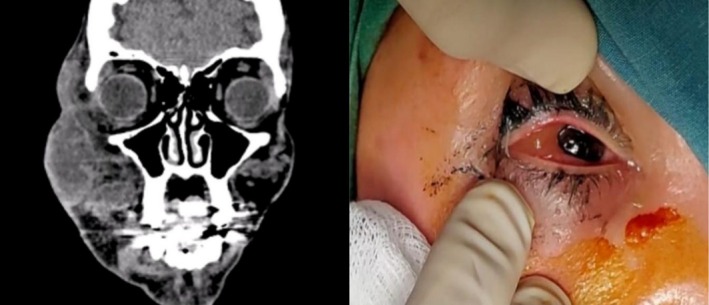
Left picture of a CT conducted and showing soft tissue thickening and fat stranding involving the right side of the face including the temporal region and orbit highly suggestive of cellulitis and orbital cellulitis. Right picture showing the intraoperative findings of the case.

The patient was taken to the operating room where incision and drainage were performed. The mucopurulent discharge was sent for culture, which later grew *Staphylococcus aureus* that was susceptible to beta‐lactam antibiotics. The patient was simultaneously started on IV ceftriaxone 1 g daily for 3 days and metronidazole 500 mg PO TID. The patient was shifted to amoxicillin and clavulanic acid 1 g PO BID for 2 weeks. The patient reported complete resolution of symptoms, as well as normalization of her labs over the next few days.

The above case demonstrates a unique case of orbital cellulitis after HA‐based filler injection. Soft‐tissue augmentation with HA fillers is a cosmetic procedure that is becoming increasingly common. These fillers are in general well tolerated and have an impressive safety profile; however orbital cellulitis is a rare side effect [1]. While periorbital cellulitis tends to affect tissues anterior to the orbital septum, leading to the absence of visual disturbances and ophthalmoplegia, orbital cellulitis affects tissues posterior to the septum leading to such symptoms. In our patient, involvement of orbital tissues and muscles alongside blurry vision strongly favors a diagnosis of orbital cellulitis over periorbital cellulitis. Facial fillers can cause visual disturbances through inflammatory, infectious, or ischemic mechanisms. These cases may occur acutely or may be delayed [2]. Sia et al. reported a case of a recurrent orbital cellulitis that presented approximately 6 years after filler injection, and culture led to the growth of *Staphylococcus aureus* , Klebsiella sp., *Proteus mirabilis* and Enterococcus faecalis [2]. Another case of a facial filler‐associated orbital cellulitis occurred 4 years after filler injection and *Staphylococcus capitis* was cultured [3]. Although *S. aureus* and *S. pyogenes* are among the most common bugs cultured in orbital cellulitis, the culture of these atypical bacteria in the cited two references may allude to an atypical pathogenesis. The cases presented in the literature report on cases of delayed orbital cellulitis, likely due to biofilm formation that lay quiescent until local trauma or immune dysregulation facilitated dispersal causing local or systemic clinical manifestations [4]. In our case, the orbital cellulitis occurred more acutely, and a more typical organism was cultured, hinting that the orbital cellulitis may have been more likely due to a direct inoculation or contiguous spread of infection from filler injection. The likelihood of occurrence of such an event may have been reduced through the use of proper safety precautions and regulations, through the aseptic non‐touch technique with pre‐planning of product, volume, and instruments and adequate cleansing techniques [5].

In conclusion, we present a rare but reported event of orbital cellulitis after soft‐tissue augmentation with facial fillers. Orbital cellulitis is a vision‐threatening emergency with high mortality and morbidity and must be recognized and treated promptly. Complications of orbital cellulitis include permanent vision loss, optic nerve damage, cavernous sinus thrombosis, meningitis, and brain abscess. Physicians must have a high index of suspicion in patients who present with swelling, erythema, pain, and ophthalmoplegia after undergoing cosmetic injections. Such infections may present acutely as in our case or may have a delayed presentation as reported in the literature.

## Consent

The authors obtained written consent from patients for their photographs and medical information to be published in print and online, with the understanding that this information may be publicly available. Patient consent forms were not provided to the journal but are retained by the authors.

## Conflicts of Interest

The authors declare no conflicts of interest.

## Data Availability

Data sharing not applicable to this article as no datasets were generated or analyzed during the current study.
